# Associations of cause-specific mortality with area level deprivation and travel time to health care in France from 1990 to 2007, a multilevel analysis

**DOI:** 10.1186/s12889-017-4562-7

**Published:** 2017-08-02

**Authors:** Walid Ghosn, Gwenn Menvielle, Stéphane Rican, Grégoire Rey

**Affiliations:** 1 INSERM, CépiDc, Epidemiological Center of Medical Causes of Death, Le Kremlin-Bicêtre, France; 2 Department of Geography, Université Paris Ouest Nanterre la Défense Laboratoire LADYSS - UMR7533, Nanterre, France; 3 Sorbonne Universités, Université Pierre et Marie Curie (Paris 6), INSERM, Institut Pierre Louis d’Epidémiologie et de Santé Publique UMRS1136, Paris, France

**Keywords:** Cause-specific mortality, Contextual association, Deprivation, Travel time to health care, Geographical mortality disparities

## Abstract

**Background:**

It is now widely accepted that social and physical environment participate in shaping health. While mortality is used to guide public health policies and is considered as a synthetic measure of population health, few studies deals with the contextual features potentially associated with mortality in a representative sample of an entire country. This paper investigates the possible role of area deprivation (FDep99) and travel time to health care on French cause-specific mortality in a proper multilevel setting.

**Methods:**

The study population was a 1% sample representative of the French population aged from 30 to 79 years in 1990 and followed up until 2007. A frailty Cox model was used to measure individual, contextual effects and spatial variances for several causes of death. The chosen contextual scale was the Zone d’Emploi of 1994 (348 units) which delimits the daily commute of people. The geographical accessibility to health care score was constructed with principal component analysis, using 40 variables of hospital specialties and health practitioners’ travel time.

**Results:**

The outcomes highlight a positive and significant association between area deprivation and mortality for all causes (HR = 1.24), cancers, cerebrovascular diseases, ischemic heart diseases, and preventable and amenable diseases (HR from 1.14 to 1.29). These contextual associations exhibit no substantial differences by sex except for premature ischemic heart diseases mortality which was much greater in women. Unexpectedly, mortality decreased as the time to reach health care resources increased. Only geographical disparities in cerebrovascular and ischemic heart diseases mortality were explained by compositional and contextual effects.

**Discussion:**

The findings suggest the presence of confounding factors in the association between mortality and travel time to health care, possibly owing to population density and health-selected migration. Although the spatial scale considered to define the context of residence was relatively large, the associations with area deprivation were strong in comparison to the existing literature and significant for almost all the causes of deaths investigated.

**Conclusion:**

The broad spectrum of diseases associated with area deprivation and individual education support the idea of a need for a global health policy targeting both individual and territories to reduce social and socio-spatial inequalities.

**Electronic supplementary material:**

The online version of this article (doi:10.1186/s12889-017-4562-7) contains supplementary material, which is available to authorized users.

## Background

Health geographic studies have demonstrated wide geographical health disparities over the last 30 years in France [1, 2]. To explain these results, the ecosocial theory explains how the health of the population is doubly influenced by the socioeconomic status of the individuals (composition) and the features of the environment of residence [3]. Hence, the related multilevel theory considers that health is also a product of the social and material environment through well-known contextual effects that are additional to individual characteristics [3, 4]. Indeed, the social and physical environment of one’s neighborhood impacts stress levels and offers various opportunities for individuals to adopt certain patterns of health behavior [5, 6]. Before examining the embodiment process which refers to how people incorporate their surrounding environment biologically, social epidemiology seeks first to identify the area features associated with deleterious consequences on health [7]. The common method of highlighting risk factors operating at several levels is to use multilevel modeling because it allows individual-level effects to be disentangled from area-level effects by measuring and taking into account the inherent correlation of individuals living in the same area of residence [8, 9]. Among key population health measures, mortality is particularly suitable for general population studies. Cause-specific mortality indicators encompass both disease incidence, which is generally considered more associated to prevention and risky behaviours, and survival of sick people, which is considered more related to health care.

Using a multilevel approach, area-level socioeconomic status has been studied in several countries as the most common contextual effect on mortality. A meta-analysis emphasized that low socioeconomic status of an area is associated with higher mortality independently of individual socioeconomic position (SEP) in many countries [10]. After adjustment of individual SEP, a higher mortality is observed in areas exhibiting high deprivation [11, 12], as well as an adverse social environment [13] and low income [14]. The effect is on average stronger when smaller geographical areas are considered [10]. In France, while individual social inequalities in mortality have been reported as widening and being among the greatest in Western Europe [15, 16], no study has yet been conducted to investigate the contextual association between social environment and mortality in a national representative sample [11, 12, 17–25].

Another feature of the physical environment that could impact health is the geographic access to health services. It is known that remote access to health care structures could constitute a barrier that discourages health care consumption [26]. To date, there is still no overview of this association with cause-specific mortality. In the literature, the distance to health care was found to be associated with the type of treatment and medical intervention [27] and also to have an adverse impact on the hepatitis C detection rate [28] and the stage of several cancers at diagnosis [29]. Ecological mortality studies have shown that a longer distance to travel to health care is significantly associated with higher mortality from asthma [30], from neonatal death after out-of hospital birth [31], in veterans with an ischemic stroke diagnosis [32], from prostate and lung cancers [33] and in emergency cases transported by ambulance [34]. Overall, this association was found to be weak. Some studies even reported that a long distance to health care is associated with lower mortality from certain causes, thus revealing the possible presence of underlying confounding factors [31, 35, 36]. In general, such ecological analyses have found that the distance to health care effect is quite sensitive to the introduction of area-level socioeconomic characteristics, thereby disregarding the evidence that these individual and contextual socioeconomic effects are additional and independent. Only one study considered the distance to hospital as a contextual effect on cancer survival in a proper multilevel analysis [37].

This paper using a multilevel analysis investigated the association between mortality by cause and two area-level features in a French general population sample. The potential contextual effects were area deprivation as a dimension of social environment and the geographic accessibility to medical care resources as part of the physical environment. In the literature, there is still no overview of these associations by cause of death. This analysis was conducted for all causes and several causes of deaths to explore the different pathways in which contextual effects may affect mortality. We also examined to what extent spatial mortality inequalities are explained by the compositional and the contextual effects to explore the potential entry points to reduce them.

## Methods

### Population data

The permanent demographic sample (EDP) is a 1/100 representative sample of the French population based on civil state declarations (births, weddings, deaths) and census forms [38]. Subjects who were at least 30 years old in 1990 and alive on 1 January 1991 were followed up for mortality until 31 December 2007. Individual characteristics and place of residence were measured at the beginning of the follow-up period from the 1990 census. Individual education was grouped into four increasing levels and corresponds to the International Standard Classification of Education (ISCED): incomplete elementary education, completed elementary education (ISCED level 1), lower secondary education (ISCED level 2), upper secondary and post secondary education (ISCED ≥3). Causes of death were obtained by indirect linkage with the French national death registry. Individuals born outside France were excluded from the analysis because of a lack of information on their vital status. Individuals aged less than 30 years were excluded to capture stable individual socio-economic characteristics (achieved level of education). Since older subjects are subject to multiple diseases, they were also excluded to prevent misclassification of causes of death.

### Causes of deaths: Classification

The underlying causes of death coded in CIM9 before 1999 and in CIM10 from 2000 were used to study cause-specific mortality. Five causes or groups of causes of death were investigated: ischemic heart diseases (IHD), cerebrovascular diseases, all cancers, amenable mortality which reflects deaths from conditions that should not occur in the presence of effective and timely health care (Amenable) and preventable deaths which could have been avoided by public health interventions focusing on wider determinants of public health, such as behavior and lifestyle factors, socioeconomic status and environmental factors (Preventable). The latter two groups of causes refer to the Office for National Statistics classification of avoidable mortality published in 2011 [39].

Cerebrovascular diseases and ischemic heart diseases were treated separately unlike cancer owing to the differing implications of the distance to care in emergency cases. ICD codes of the groups of causes of death are detailed in the Additional file 1: Table S1. Standard mortality ratios (SMR) adjusted on age and sex were calculated for each individual and contextual variables (Table 1).

**Table 1:** Distribution of population, deaths and SMR across individual and area-level factors on 1st January 1990

Variables	All					Women					Men				
	population	(%)	deaths	(%)	SMR	population	(%)	deaths	(%)	SMR	population	(%)	deaths	(%)	SMR
Individual Education															
Upper and post-secondary	56,142	(21.7)	6176	(11.3)	0.71	27,277	(20)	1972	(8.3)	0.75	28,865	(23.6)	4204	(13.6)	0.69
Lower secondary	72,308	(27.9)	9824	(18)	0.93	34,380	(25.2)	3551	(15)	0.90	37,928	(31)	6273	(20.3)	0.94
Completed elementary	67,483	(26.1)	18,088	(33.1)	0.99	39,552	(29)	8258	(34.9)	0.96	27,931	(22.8)	9830	(31.8)	1.01
Incompleted elementary	63,086	(24.4)	20,507	(37.6)	1.21	35,368	(25.9)	9882	(41.8)	1.17	27,718	(22.6)	10,625	(34.3)	1.25
Area deprivation															
Q1 (least deprived)	51,361	(19.8)	9186	(16.8)	0.91	27,766	(20.3)	4280	(18.1)	0.94	23,595	(19.3)	4906	(15.9)	0.88
Q2	52,390	(20.2)	10,477	(19.2)	0.96	27,836	(20.4)	4564	(19.3)	0.96	24,554	(20.1)	5913	(19.1)	0.97
Q3	50,970	(19.7)	10,994	(20.1)	1.00	26,781	(19.6)	4619	(19.5)	0.98	24,189	(19.8)	6375	(20.6)	1.02
Q4	51,911	(20)	11,609	(21.3)	1.03	26,979	(19.8)	4925	(20.8)	1.03	24,932	(20.4)	6684	(21.6)	1.03
Q5	52,387	(20.2)	12,329	(22.6)	1.09	27,215	(19.9)	5275	(22.3)	1.09	25,572	(20.9)	7054	(22.8)	1.09
Time to health care															
Q1 (shortest travel time)	54,781	(21.1)	10,791	(19.8)	0.96	29,990	(22)	5071	(21.4)	0.99	24,791	(20.2)	5720	(18.5)	0.95
Q2	50,070	(19.3)	9464	(17.3)	0.98	26,647	(19.5)	4131	(17.5)	0.98	23,423	(19.1)	5333	(17.2)	0.97
Q3	50,179	(19.4)	10,629	(19.5)	1.04	26,248	(19.2)	4471	(18.9)	1.03	23,931	(19.5)	6158	(19.9)	1.04
Q4	51,924	(20)	11,590	(21.2)	1.04	27,088	(19.8)	4847	(20.5)	1.02	24,836	(20.3)	6743	(21.8)	1.06
Q5	52,065	(20.1)	12,121	(22.2)	0.98	26,604	(19.5)	5143	(21.7)	0.99	25,461	(20.8)	6978	(22.6)	0.98

Deaths: individuals who have died before 31st December 2007 inclusive

*SMR* Standard Mortality Rate, *Q* ordered population-weighted quintile

### Spatial scale

The Zone d’Emploi scale in 1994 (ZE94, 348 units) was considered as the contextual level (level 2) for several reasons. It was built on daily commute so it draws the boundaries of the context in which people live. In addition, the significance of the contextual effect and the spatial variance estimations are reliable at this scale. There was a minimum of 86 people, a maximum of 7952 people with a median population of 464 inhabitants and a mean population of 760.

### Area-level deprivation

The French deprivation index FDep99 for the year 1999 (middle of the follow-up period) was used to measure the social environment. This score is defined as the first component of a principal component analysis, weighted by population size, of four socio-economic ecological variables that are not redundant, and which are routinely collected: percentage of high-school graduates, median household income, percentage of blue-collar workers and the unemployment rate. The underlying interpretation of this index is that deprivation is seen as an accumulation of disadvantages and it is built relatively to the population exposure. Population-weighted quintiles of this index were used to allow the association to be non-linear (non-log linear to be more specific).

### Travel time to health services

Travel time to health practitioners and hospital specialties in 2007 on the commune scale (smallest administrative scale, 36,000 units) were provided by IRDES (Institute for research and information on health economics). Coldefy et al. estimated the travel time with the ChronoMap© software on the basis of travel time between the geometric center of each commune to the nearest commune in which the concerned health care resources were available. The methodology is detailed in the IRDES working document [40]. To summarize the geographical accessibility to global health care resources, the first component of a principal component analysis of all hospital specialties (23 variables) and health practitioners’ (17 variables) travel time was first calculated on the commune scale (70% of the total variance explained). Then, the population-weighted mean of this score was calculated on the ZE94 scale. This score reflects an average time one must travel to reach global health care resources. In a multilevel framework, it is considered as a physical characteristic of the environment, an opportunity shared by the inhabitants that is spatially differentiated as intra-geographical unit mortality differentials can not be explained by the tiny inter-individual disparities in time to travel to reach health care. Population-weighted quintiles of this score were considered for statistical analysis. The list of health care resources is detailed in the Additional file 1: Table S2.

### Statistical analysis

Proportional hazard two-level Cox models (also known as frailty models) were used to estimate the associations between mortality and individual/contextual factors. The exogenous variables introduced in the models were sex, individual education (level 1), the quintile of deprivation of the area of residence and the quintile of mean travel time to health care resources (level 2). The considered random effects followed a centered Gaussian distribution. The distribution’s variance of this random effect reflected the spatial variability of mortality hazard. All the estimations and procedures were executed with R [41] using the coxme package [42, 43] and significance level was fixed at 0.05. The contextual associations with deprivation and travel time to health care were estimated by sex for all ages under 65 years and for people aged 65 and older in separate models. When the trend of these associations is exposed, it corresponds to the log-linear effect multiplied by the mean score difference between the fifth and first quintile.

In order to summarize and compare spatial mortality differentials before and after adjustment on individual and then on contextual factors, mortality ratios were calculated between the tenth and first decile deduced from the Gaussian distribution with these corresponding estimated spatial variances..

## Results

There was a fairly balanced distribution of the population between men and women according to individual and contextual explanatory variables, except for the two lowest education categories where women were overrepresented (Table 1). The largest mortality gradient was observed for the individual education categories between the least and the most educated categories with larger differences for men. Among the area level variables, mortality inequalities by area deprivation category were larger than for travel time to health care categories and were larger for men than women. There was also an inverse U-shaped mortality relationship with time to health care quintiles. Travel time to health care increased with mortality, except for the longest travel time areas where mortality was close to the average (Table 1).

In the separate models, there was a strong individual education gradient in mortality measured by HR which varied from 1.31 to 1.75 for technical high school level and individuals with no education, respectively (Model 2). Mortality was much lower in women than in men in all models. There was a positive and significant association between area deprivation and mortality (Model 3) but no association (*p*-value = 0.29) with travel time to health care services (Model 4). In the model with individual education and area deprivation, the association with individual education was not affected although the association with area deprivation was halved but remained significant (Model 5). The introduction of individual education or area deprivation made the association between mortality and travel time to health care negative and significant though very weak (Model 6 and 7). Globally in the model with travel time to health care variable, the association with area deprivation was magnified while the individual education associations remained the same in all adjustments. In the full model, individual education and area deprivation were positively associated with mortality while the association with travel time was negative, significant and stronger than in the other models. All the interactions between area-level and individual factors were tested and were not significant (results not shown).

Spatial variance decreased 11% when individual education was introduced compared to Model 1, 21% with area deprivation only and 5% with the presence of travel time only. In the models with at least two variables, individual education and area deprivation explained 16% of the spatial variations, individual education and travel time explained 18%, while area deprivation and travel time reduced 40% of total variations, which was nearly to the same extent as in the full model (Model 8) (Table 2).

**Table 2:** Association between all-cause mortality and sex, individual education, contextual deprivation and time to health care

Variables	Model 1	Model 2	Model 3	Model 4	Model 5	Model 6	Model 7	Model 8
	HR	HR	HR	HR	HR	HR	HR	HR
Individual effects								
Men	ref	ref	ref	ref	ref	ref	ref	ref
Women	0.49***	0.48***	0.49***	0.49***	0.48***	0.48***	0.49***	0.48***
Upper and post-secondary		ref			ref	ref		ref
Lower secondary		1.31***			1.31***	1.31***		1.31***
Completed elementary		1.42***			1.42***	1.42***		1.42***
Incompleted elementary		1.75***			1.74***	1.75***		1.75***
Contextual Effects								
Area Deprivation								
Q1 (least deprived)			ref		ref		ref	ref
Q2			1.05		1.01		1.08*	1.05
Q3			1.08**		1.02		1.14***	1.09**
Q4			1.13***		1.04		1.19***	1.13***
Q5			1.17***		1.06*		1.26***	1.17***
Trend			1.10***		1.05***		1.15***	1.10***
Travel time to health care								
Q1 (shortest time)				ref		ref	ref	ref
Q2				0.99		0.97	0.96	0.95
Q3				1.04		0.98	0.96	0.93*
Q4				1.05		0.98	0.94	0.91**
Q5				0.98		0.91**	0.87***	0.84***
Trend				0.98		0.93***	0.89***	0.87***
Spatial Variance (ZE94)	0.013	0.011	0.010	0.012	0.011	0.010	0.008	0.008

The Trend is estimated from a separate model in which the concerned contextual effect has been introduced as a linear effect (log-linear). The displayed trend effect is the linear effect multiplied by the mean score difference between the fifth and the first quintile

*HR* Hazard Ratio. *Q* ordered population-weighted quintile

*p-value <0.050, **p-value <0.010, ***p-value <0.001

All the associations had the same direction as for all-cause mortality except travel time with cerebrovascular disease mortality, which was positive but not significant. The association with individual education and contextual deprivation were both positive and significant but weakest for all tumors and strongest for preventable disease mortality. The association with travel time to health care was negative, significant and strongest for preventable diseases. Comparing the spatial variances of the fully adjusted models, the lowest estimates were obtained for cerebrovascular mortality and the highest ones were obtained for preventable and amenable diseases (Table 3).

**Table 3:** Association between cause-specific mortality and sex, individual education, contextual deprivation and time to health care

Variables	Cerebrovascular Diseases		Ischemic Heart Diseases	All Tumors	Amenable Diseases	Preventable Diseases
	HR		HR	HR	HR	HR
Men	ref		ref	ref	ref	ref
Women	0.61***		0.37***	0.43***	0.59***	0.31***
Individual effects						
Upper and post-secondary	ref		ref	ref	ref	ref
Lower secondary	1.27***		1.30***	1.28***	1.26***	1.44***
Completed elementary	1.41***		1.46***	1.29***	1.39***	1.57***
Incompleted elementary	1.71***		1.62***	1.47***	1.72***	1.99***
Contextual Effects						
Area Deprivation						
Q1 (least deprived)	ref		ref	ref	ref	ref
Q2	1.06		1.11	1.04	1.05	1.04
Q3	1.08		1.14*	1.10*	1.08	1.13*
Q4	1.07		1.22**	1.12**	1.11*	1.13*
Q5	1.21**		1.29***	1.14***	1.20***	1.24***
Trend	1.10**		1.15***	1.07***	1.12***	1.13***
Travel time to health care						
Q1 (shortest time)	ref		ref	ref	ref	ref
Q2	0.98		0.88*	0.98	0.93	0.94
Q3	1.15*		1.04	0.90**	0.97	0.93
Q4	1.11		1.01	0.90**	0.93	0.92
Q5	1.05		0.90	0.83***	0.88**	0.80***
Trend	0.98		0.95*	0.93***	0.95***	0.91***
Spatial Variance (ZE94)	0.003		0.007	0.006	0.010	0.017

The Trend is estimated from a separate model in which the concerned contextual effect has been introduced as a linear effect (log-linear). The displayed trend effect is the linear effect multiplied by the mean score difference between the fifth and the first quintile

*HR* Hazard Ratio, *Q* ordered population-weighted quintile

*p-value <0.050, **p-value <0.010, ***p-value <0.001

Deprivation was significantly associated to almost the same extent with all-cause mortality regardless of age and sex (Table 3). Considering cause-specific mortality, the positive association between contextual deprivation and mortality was slightly stronger for men than for women except for ischemic heart disease. For men, the association with deprivation was a little stronger for premature mortality than for older persons, not significant for premature and older cerebrovascular diseases and globally slightly stronger for preventable diseases and IHD, but these differences remained very small. For women, the association by age with deprivation was contrasted. It was the strongest for premature mortality by ischemic heart disease while the associations with cerebrovascular diseases, cancers and preventable diseases were not statistically significant. For older women, contextual deprivation was associated with all the causes considered except for cancers. The association with travel time to health care was always negative except for women’s premature mortality by cerebrovascular disease, although it was not significant. There were no clear differences by sex while the association was much stronger by cardiovascular disease mortality in men under 65 years of age (Table 4).

**Table 4:** Contextual associations with cause-specific mortality by sex and age

Causes of Deaths	All ages		< 65 years		> = 65 years	
	Time to HC	Deprivation	Time to HC	Deprivation	Time to HC	Deprivation
Men						
All causes	0.93***	1.10***	0.91***	1.09^**^	0.95***	1.10***
Cerebrovascular diseases	0.97	1.11*	0.88	1.26	0.99	1.08
Ischemic Heart Disease	0.94*	1.13**	0.89*	1.18*	0.96	1.11*
Cancers	0.91***	1.10***	0.88***	1.14***	0.94***	1.08**
Amenable Diseases	0.94**	1.12***	0.91*	1.15**	0.95**	1.12***
Preventable Diseases	0.91***	1.14***	0.88***	1.15***	0.93***	1.13***
Women						
All causes	0.94***	1.11***	0.92**	1.09*	0.94***	1.11***
Cerebrovascular diseases	1.00	1.10*	1.08	0.98	0.99	1.11*
Ischemic Heart Disease	0.98	1.18***	1.00	1.52*	1.00	1.17**
Cancers	0.94***	1.02	0.89**	1.05	0.96*	1.01
Amenable Diseases	0.96**	1.11***	0.95	1.16**	0.96**	1.11***
Preventable Diseases	0.93**	1.09**	0.84***	1.05	0.95*	1.09*

All the outcomes result from models fully adjusted on individual education, age and sex. They are expressed as the log-linear effect between the fifth (Q5) and first quintile (Q1) means difference of each area-based measure

*Time to HC*: travel time to Health Care structures

*p-value <0.050, ** *p*-value <0.010*** *p*-value <0.001

**Fig. 1:**
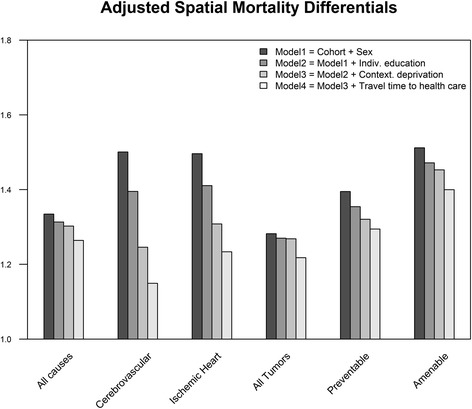
Spatial Mortality Differentials by Cause adjusted for individual education, area deprivation and travel time to health care. Deciles are extracted from spatial mortality distribution estimated by spatial variance term for each model

When adjusted on sex and cohort, spatial mortality differences were greater for cerebrovascular diseases, IHD and amenable diseases (Fig. 1). When adjusted on educational composition, cerebrovascular and ischemic heart disease inter-decile mortality differences dropped from nearly 1.5 to 1.4 while all other causes were little affected. The introduction of area deprivation made the mortality ratio of the same two causes drop to nearly 1.25. The effect of travel time to health care explained the greatest part of each mortality spatial variance for all-cause, all tumors and amenable diseases (Fig. 1).

## Discussion

In this study, area deprivation had a positive and significant contextual effect that was additional to individual education for all the causes of death investigated. There was no clear difference between men and women’s associations except for premature IHD and all-cancer mortality which were respectively stronger and weaker for women than for men. On the contrary, the association with travel time to health care was unexpected: mortality was higher in areas close to health care resources and decreased with longer travel time, exhibiting significant negative trends for all causes of deaths investigated except for cerebrovascular disease mortality. This negative association with mortality was dependent on the introduction of educational composition or area deprivation. Similarly, the introduction of travel time to health care resources into the individual and collective socio-economic level adjusted model reinforced the contextual effects of deprivation on all-cause mortality. In all models, the individual education effect remained significant, its magnitude was the largest and it was never modified by the introduction of any area-level factor. The spatial differentials were largely explained by the individual and contextual characteristics investigated for cerebrovascular diseases and IHD mortality. Each factor explained almost the same amount of spatial disparity for these causes while a very small proportion was explained for other causes of death.

### Comparison with existing results

This work confirms the existence of a contextual effect of deprivation on mortality for several causes of death. In comparison to other studies, the magnitude of this effect is among the strongest documented to date [11, 12] although it was expected to be weaker, as reported in studies using a larger scale of area definition [10]. A multilevel study in the Norwegian general population also reported a significant negative association between cancer survival and area-level socioeconomic status [37] which is in line with our results. The few multilevel studies investigating the contextual association between distance to health care and mortality make these comparisons difficult. Aside from these limitations, an ecological study reported a reverse association with heart disease mortality after the introduction of socioeconomic characteristics [35], a shift that was also observed in the present study. Distance to health care affected the survival of American veterans experiencing acute ischemic stroke [32]. The latter result is compatible with the positive although not significant association with cerebrovascular disease mortality in women under 65 years of age in our study. However, the veterans’ study was not adjusted on individual and contextual socioeconomic characteristics and travel time to hospital is on average far longer in the USA than in France [44]. In France, a positive and significant association was found between distance to medical care and survival of patients with some digestive cancers [45]. However, our findings cannot confirm this result owing to a lack of statistical power and differing key methodological choices (other deprivation index, no adjustment on individual SEP, finer scale of analysis, a population that is largely rural).

### Interpretation

The individual educational effect on mortality is likely to be mediated by other socioeconomic characteristics. Poor education affects material resources through lower income and higher risk of unemployment, and it increases the likelihood of doing a tedious job and being exposed to psychosocial risk factors [46]. It could also constitute a possible gateway to deleterious health behaviors such as smoking, alcohol abuse, lower fruit and vegetable consumption and lower health care utilization, implying late-stage diagnosis of chronic diseases [47–49]. In comparison to contextual effects, individual education effects were largely stronger for all the causes of death investigated.

The contextual effect of area deprivation in the literature is related to greater access to fast food, alcohol and tobacco retailers, a noisier and more violent environment, higher exposure to air pollution and globally to features that contribute to increasing stress levels and offering more opportunities to adopt behaviors with adverse health effects [5, 10, 50–53]. However, the Zone d’Emploi scale is rather large compared to the census or ward scale. The variation in the density of alcohol and tobacco retailers and food stores cannot solely explain the association with contextual deprivation. The interpretation might lie more in local constructions of lifestyle and cultural factors. Apart from these multifactorial explanations that require more investigation in France, it is also possible that the healthy migrant effect eventually increases the relative mortality level of more deprived areas as they become more segregated. Indeed, if healthy people leave deprived areas, the mortality of these zones could be inflated because of the resulting poorer health of the remaining sedentary population without the presence of any area-level effect.

Concerning the effect of travel time to health care resources, the negative and globally weak association makes it hard to interpret. It is unlikely that a longer distance to travel to health services increases the consumption of health care. Regarding the initial hypothesis, this inverse association seems artefactual. It is likely to be confounded by the higher mortality in urban than in rural areas once area deprivation is controlled for. The urbanity gradient encompasses several area features that are highly correlated, which makes it difficult to decompose the risk factors of mortality (the correlation with density was −0.45 on the ZE94 scale), as the introduction of population density made this association positive but not significant (see Additional file 1: Table S3). However, the notion of urban and rural space is fuzzy at this level of analysis. Another potential interpretation for this association is reverse causation. It is possible that people with higher needs of health care live or move closer to medical care services while those with a lower risk live farther away. In the literature, the renouncement of care is motivated more by financial obstacles than travel time needed to reach health care services, as reported for the Nord-Pas-de-Calais region [54]. Therefore, one could expect to find most of the differences in health due to renouncement to health care in social health inequalities [55]. The concomitant compositional and contextual explanation of spatial mortality disparities by cerebrovascular disease and ischemic heart disease means that these factors are a key gateway to reducing them on this scale.

### Limitations

In this work, the Zone d’emploi scale was considered as being constitutive of a living environment as a whole since the working population commutes daily within these boundaries. Although much of the spatial dispersion of mortality and about 40% of the area deprivation differentials [56] were diluted because of aggregation of the communes within the Zone d’Emploi scale, the spatial variance and the associated contextual effects could be reliably assessed. In addition, the estimations at commune level (36,000 units obtained without a multilevel model) were found to point in the same direction with the same magnitude except for a larger adverse effect of contextual deprivation (results not shown), thus confirming previous findings [10]. The score of geographical accessibility to health care reflects both hospital and ambulatory medicine which do not share the same underlying link to mortality. As the spatial correlations between specialties were very high (even on a finer scale), the travel time variable was first estimated at the commune level and then aggregated on the Zone d’emploi scale as the average travel time score per inhabitant to reach global health care services. Again, much information was diluted. A serious limitation of the travel time score lies in the lack of information on the population demand for care, the volume of practitioners and hospital activity. These features have to be taken into consideration in a further study by evaluating both the varying contribution on demand that is imputable to population socio-demographic characteristics and the catchment area of health care structures by using the enhanced two-step floating catchment area method. [57]. However, while the practitioner’s volume of activity can be measured, demand for care does not always reflect the need for health care. This is particularly true for socially disadvantaged populations who first consult a general practitioner and rarely consult specialists in comparison to socially advantaged people [55]. Further studies should also take into account the waiting time to obtain a consultation, which can constitute a potential barrier to health care. Another limitation relative to the travel time data is that they concerned 2007, which was relatively late in comparison to the beginning of the follow-up. However, the correlations between the distances (in km) to health care in 1990 and 2007 were very high (from 0.97 to 0.99) which confirms that no major change had occurred at this scale of analysis.

The causes of death categories considered aggregated several diseases that do not necessarily share the same risk factors. However, the amenable and preventable diseases categories define deaths that could have been avoided respectively by appropriate medical care and better access to preventive care [39]. Therefore these categories of causes of death are likely to be influenced by the physical and environmental area-level features investigated.

Another limitation regarding the magnitude of spatial variance is that it may result from an unmeasured residual compositional effect. On the other hand, contextual effects could have been masked when adjusting on individual characteristics which mediate the impact of the context on mortality [13, 58, 59].

## Conclusion

This study is the first to assess a contextual effect of deprivation on mortality in France in the general population. After adjustment on individual SEP and area deprivation, there were still large spatial mortality differences between the Zones d’Emploi. This implies that individual education cannot explain spatial mortality inequalities on this scale. Nor was there any evidence of a deleterious effect of travel time to health care services on mortality. Further work should not focus only on geographical accessibility but rather on a composite measure of accessibility that combines distance, availability of health care providers and health care needs of the population. Several spatial factors that are associated with urban density remain also to be investigated. The mediating processes linking contextual deprivation to higher mortality also deserve further attention. The substantial spatial variability in mortality after adjustment on individual and contextual socioeconomic data suggests that contextual features could impact health. Finally, beyond the need for a national focus on individuals with low educational attainment, targeting deprived areas for public health interventions is a key issue for reducing social mortality inequalities.

## Additional file

Additional file 1: Table S1. ICD codes of the causes of death investigated. **Table S2**. Services and specialties included in the travel time to health care score. **Table S3**. Association between cause-specific mortality and sex, individual education, contextual deprivation, time to health care and population density. (DOCX 25 kb)

## Abbreviations

HC: Health care
HR: Hazard ratio
IHD: Ischemic heart disease
ISCED: International Standard Classification of Education
SEP: Socio-economic position
ZE94: Zone d’Emploi scale of 1994
