# Supplementary material for: Associations of cause-specific mortality with area level deprivation and travel time to health care in France from 1990 to 2007, a multilevel analysis
Source: BMC Public Health. 2017 Aug 2;18:86. doi: 10.1186/s12889-017-4562-7 (PMC5540569; doi:10.1186/s12889-017-4562-7)
Supplement: Additional file 1: Table S1. — ICD codes of the causes of death investigated. Table S2. Services and specialties included in the travel time to health care score. Table S3. Association between cause-specific mortality and sex, individual education, contextual deprivation, time to health care and population density. (DOCX 25 kb) [file 12889_2017_4562_MOESM1_ESM.docx]

**Table S1: ICD codes of the causes of death investigated**

| **Causes of deaths** | **CIM9** |  | **CIM10** |
| --- | --- | --- | --- |
| Ischemic Heart Diseases | 410-414 |  | I20-I25 |
| Cerebrovascular Diseases | 430-438 |  | I60-I69 |
| Cancers | 140-239 |  | C00-D48 |
|  |  |  |  |
| **Amenable mortality** |  |  |  |
| Intestinal infectious diseases | 001-009 |  | A00-A09 |
| Tuberculosis | 010-018,137 |  | A15-A19, B90 |
| Other infections | 037,032,045 |  | A35-A36, A80 |
| Whooping cough | 033 |  | A37 |
| Selected invasive bacterial and protozoal infections | 034,035,036,038,4828, 084,3200-3205,3208, 3209,322,682 |  | A38-A41, A46, A481, B50-B54, G00, G03, L03 |
| Measles | 55 |  | B05 |
| Malignant neoplasm of colon and rectum | 153-154 |  | C18-C21 |
| Malignant melanoma of skin | 172 |  | C43 |
| Other malignant neoplasms of skin | 173 |  | C44 |
| Malignant neoplasms of breast | 174-175 |  | C50 |
| Malignant neoplasm of cervix uteri | 180 |  | C53 |
| Malignant neoplasm of corpus uteri and uterus unspecified | 179,182 |  | C54-C55 |
| Malignant neoplasm of testis | 186 |  | C62 |
| Malignant neoplasm of bladder | 188 |  | C67 |
| Malignant neoplasm of thyroid gland | 193 |  | C73 |
| Hodgkin’s disease | 201 |  | C81 |
| Leukaemia | 204-208 |  | C91-C95 |
| Benign neoplasms | 210-229 |  | D10-D36 |
| Disorders of thyroid gland | 240-246 |  | E00-E07 |
| Diabetes mellitus | 250 |  | E10-E14 |
| Epilepsy and status epilepticus | 345 |  | G40-G41 |
| Rheumatic and other valvular heart disease | 391,3920,393-398 |  | I01-I09 |
| Hypertensive diseases | 401-405 |  | I10-I15 |
| Ischaemic heart disease | 410-414 |  | I20-I25 |
| Cerebrovascular diseases | 430-438 |  | I60-I69 |
| Other respiratory | 460-466,470-478 494,500-508, 510-519 |  | J00-J08, J20-J39,  J47-J99 |
| Influenza (including swine flu) | 487 |  | J09-J11 |
| Pneumonia | 480-486 |  | J12-J18 |
| Chronic Obstructive Pulmonary Disorder | 490-492,496 |  | J40-J44 |
| Asthma | 493 |  | J45-J46 |
| Gastric and duodenal ulcer | 531-534 |  | K25-K28 |
| Acute abdomen, appendicitis, intestinal obstruction,  cholecystitis / lithiasis, pancreatitis, hernia | 540-543,550-553, 574-577 |  | K35-K38, K40-K46, K80-K83, K85-K86, K91.5 |
| Nephritis and nephrosis | 580-583,584,586, 588,589 |  | N00-N07, N17-N19, N25, N27 |
| Obstructive uropathy & prostatic hyperplasia | 593,5920,5921, 598,600 |  | N13, N20-N21, N35, N40, N99.1 |
| Pregnancy, childbirth and the puerperium | 630-677 |  | O00-O99 |
| Complications of perinatal period | 760-779 |  | P00-P96, A33 |
| Congenital malformations, deformations and chromosomal anomalies | 740-759 |  | Q00-Q99 |
| Misadventures to patients during surgical and medical care | 870-876 |  | Y60-Y69, Y83-Y84 |
|  |  |  |  |
| **Preventable mortality** |  |  |  |
| Tuberculosis | 010-018,137 |  | A15-A19, B90 |
| Other infections | 037,032,045 |  | A35-A36, A80 |
| Whooping cough | 33 |  | A37 |
| Viral hepatitis | 70 |  | B15-B19 |
| HIV/AIDS | 42 |  | B20-B24 |
| Malignant neoplasm of lip, oral cavity and pharynx | 140-149 |  | C00-C14 |
| Malignant neoplasm of oesophagus | 150 |  | C15 |
| Malignant neoplasm of stomach | 151 |  | C16 |
| Malignant neoplasm of liver | 155 |  | C22 |
| Malignant neoplasm of trachea, bronchus and lung | 162 |  | C33-C34 |
| Malignant melanoma of skin | 172 |  | C43 |
| Other malignant neoplasms of skin | 173 |  | C44 |
| Alcohol related diseases, excluding external causes | 291,303,3050,3575, 4255,5353,5710-5713 |  | F10, G312, G621,  I426, K292, K70, K73, K741, K742, K746, K747, K748, K749, K860 |
| Illicit drug use disorders | 304-305 |  | F11-F16, F18-F19 |
| Ischaemic heart disease | 410-414 |  | I20-I25 |
| DVT with pulmonary embolism | 415,4511-4513,4519,4539 |  | I26, I80.1-I80.3, I80.9, I82.9 |
| Aortic aneurysm and dissection | 441 |  | I71 |
| Influenza (including swine flu) | 487 |  | J09-J11 |
| Pneumonia | 480-486 |  | J12-J18 |
| Chronic Obstructive Pulmonary Disorder | 490-492,496 |  | J40-J44 |
| Transport Accidents | 800-848,9290,9291 |  | V01-V99 |
| Accidental Injury | 880-888,910,850-869, 870-879,890-909, 911-928,9292-9299 |  | W00-X59 |
| Suicide and self inflicted injuries | 950-959 |  | X60-X84, Y10-Y34 |
| Homicide/ Assault | 960-969 |  | X85-Y09, U50.9 |

**Table S2: Services and specialties included in the travel time to health care score**

| **Ambulatory medicine** | **Hospital services** |
| --- | --- |
| General practitioner | Hepato-Gastro-Enterology |
| Nurse | Nephrology |
| Dentist | Digestive surgery |
| Complementary and alternative medicine (GP) | Haematology |
| Ophtalmologist | Pulmonology |
| Radiologist | Endocrinology |
| Physiotherapist | Endoscopy |
| Gynaecologist | Neurology |
| Cardiologist | Orthopedic surgery |
| Dermatologist | Ophtalmology |
| Psychiatrist | Urology |
| Paediatrician | Cardiology |
| Otolaryngologist | Rheumatologiy |
| Gastroentorologist | Dermatology |
| Rheumatologist | Vascular surgery |
| Pulmonologist | Thoracic surgery |
| Urologist | Maternity level 2 |
|  | Maternity level 3 |
|  | Neurosurgery |
|  | Cardiac surgery |
|  | Otolaryngologist |
|  | Maternity level 1 |
|  | Severly burned patients |

**Table S3: Association between cause-specific mortality and sex, individual education, contextual deprivation, time to health care and population density**

| **Variables** | **All Causes** | | **Ischemic Heart**  **Diseases** | | **Cerebrovascular**  **Diseases** | | **All Tumors** | | **Preventable**  **Diseases** | | **Amenable**  **Diseases** | |
| --- | --- | --- | --- | --- | --- | --- | --- | --- | --- | --- | --- | --- |
|  |  |  |  |  |  |  |  |  |  |  |  |  |
|  | **HR** |  | **HR** |  | **HR** |  | **HR** |  | **HR** |  | **HR** |  |
| *Individual effects* |  |  |  |  |  |  |  |  |  |  |  |  |
| Men | *ref* |  | *ref* |  | *ref* |  | *ref* |  | *ref* |  | *ref* |  |
| Women | 0.48 | *** | 0.37 | *** | 0.61 | *** | 0.43 | *** | 0.31 | *** | 0.59 | *** |
| Upper and post-secondary | *ref* |  | *ref* |  | *ref* |  | *ref* |  | *ref* |  | *ref* |  |
| Lower secondary | 1.31 | *** | 1.30 | *** | 1.26 | *** | 1.28 | *** | 1.44 | *** | 1.26 | *** |
| Incomplete Elementary | 1.42 | *** | 1.46 | *** | 1.42 | *** | 1.29 | *** | 1.57 | *** | 1.39 | *** |
| Incomplete Elementary | 1.75 | *** | 1.62 | *** | 1.71 | *** | 1.47 | *** | 1.99 | *** | 1.72 | *** |
| *Contextual effects* |  |  |  |  |  |  |  |  |  |  |  |  |
| Deprivation |  |  |  |  |  |  |  |  |  |  |  |  |
| Q1 (least deprived) | *ref* |  | *ref* |  | *ref* |  | *ref* |  | *ref* |  | *ref* |  |
| Q2 | 1.09 | ** | 1.14 | * | 1.10 |  | 1.07 | * | 1.08 |  | 1.09 | * |
| Q3 | 1.12 | *** | 1.15 | * | 1.09 |  | 1.11 | ** | 1.16 | ** | 1.10 | * |
| Q4 | 1.16 | *** | 1.23 | ** | 1.08 |  | 1.13 | *** | 1.17 | ** | 1.14 | ** |
| Q5 | 1.21 | *** | 1.31 | *** | 1.23 | ** | 1.15 | *** | 1.27 | *** | 1.23 | *** |
| Travel ttime to health care |  |  |  |  |  |  |  |  |  |  |  |  |
| Q1 (shortest travel time) | *ref* |  | *ref* |  | *ref* |  | *ref* |  | *ref* |  | *ref* |  |
| Q2 | 1.00 |  | 0.92 |  | 1.07 |  | 1.04 |  | 1.00 |  | 0.97 |  |
| Q3 | 1.04 |  | 1.13 |  | 1.35 | *** | 1.02 |  | 1.06 |  | 1.08 |  |
| Q4 | 1.06 |  | 1.16 |  | 1.38 | ** | 1.06 |  | 1.10 |  | 1.09 |  |
| Q5 | 1.00 |  | 1.02 |  | 1.32 | ** | 0.97 |  | 0.98 |  | 1.03 |  |
| Population density |  |  |  |  |  |  |  |  |  |  |  |  |
| Q1 (least dense) | *ref* |  | *ref* |  |  |  |  |  |  |  |  |  |
| Q2 | 1.02 |  | 0.97 |  | 1.00 |  | 0.99 |  | 1.01 |  | 1.00 |  |
| Q3 | 1.09 | *** | 1.07 |  | 1.08 |  | 1.04 |  | 1.10 | * | 1.09 | * |
| Q4 | 1.18 | *** | 1.13 |  | 1.21 | * | 1.16 | *** | 1.21 | *** | 1.17 | *** |
| Q5 | 1.24 | *** | 1.15 |  | 1.30 | * | 1.21 | *** | 1.28 | *** | 1.22 | ** |
| Spatial variance (ZE94) | 0.007 |  | 0.005 |  | 0.000 |  | 0.003 |  | 0.014 |  | 0.008 |  |
